# Autism spectrum disorder: prospects for treatment using gene therapy

**DOI:** 10.1186/s13229-018-0222-8

**Published:** 2018-06-20

**Authors:** Matthew Benger, Maria Kinali, Nicholas D. Mazarakis

**Affiliations:** 1 Gene Therapy, Centre for Neuroinflammation and Neurodegeneration, Division of Brain Sciences, Faculty of Medicine, Imperial College London, Hammersmith Hospital Campus, W12 0NN, London, UK; 2 Present address: The Portland Hospital, 205-209 Great Portland Street, London, W1W 5AH UK

**Keywords:** Autistic spectrum disorder, Synaptic dysfunction, ASD models, Gene therapy, Viral vector

## Abstract

Autism spectrum disorder (ASD) is characterised by the concomitant occurrence of impaired social interaction; restricted, perseverative and stereotypical behaviour; and abnormal communication skills. Recent epidemiological studies have reported a dramatic increase in the prevalence of ASD with as many as 1 in every 59 children being diagnosed with ASD. The fact that ASD appears to be principally genetically driven, and may be reversible postnatally, has raised the exciting possibility of using gene therapy as a disease-modifying treatment. Such therapies have already started to seriously impact on human disease and particularly monogenic disorders (e.g. metachromatic leukodystrophy, SMA type 1). In regard to ASD, technical advances in both our capacity to model the disorder in animals and also our ability to deliver genes to the central nervous system (CNS) have led to the first preclinical studies in monogenic ASD, involving both gene replacement and silencing. Furthermore, our increasing awareness and understanding of common dysregulated pathways in ASD have broadened gene therapy’s potential scope to include various polygenic ASDs. As this review highlights, despite a number of outstanding challenges, gene therapy has excellent potential to address cognitive dysfunction in ASD.

## Background

“Between stimulus and response there is a space. In that space is our power to choose our response. In our response lies our growth and our freedom”—Viktor E Frankl.

In autism spectrum disorder (ASD), a neurodevelopmental disorder affecting ~ 1.5% of the population [1], aetiologically diverse deficits in cognitive plasticity lead to broad impairments in communication and restricted, repetitive behaviours [2]. Comorbidities are common (~ 70% of cases) and include epilepsy; attention, mood and language disorders; sleep disturbance; gastrointestinal problems; and intellectual disability [3].

Despite the great personal and sociological cost of ASD (estimated to be $2 million/patient/year [4]), only the antipsychotics risperidone and aripiprazole are currently FDA-approved to treat ASD, indicated solely in the treatment of irritability symptoms [5]. A fundamental reason for this lack of disease-modifying therapies may relate to ASD’s pathogenesis, which appears to be principally driven by heterogeneous genetic mutations and variants and modulated by diverse gene × environment interactions, to include pregnancy-related factors (e.g. maternal immune activation, maternal toxins) and perinatal trauma [2, 6–10]. Many of the encoded proteins implicated in ASD pathogenesis—such as cytoskeletal proteins, cell adhesion molecules and DNA-binding proteins—may be ‘undruggable’ using conventional small molecule drugs, which principally only modulate the function of receptors and enzymes [11].

In contrast, gene therapy—broadly defined as the delivery of nucleic acid polymers into cells to treat disease—may be used to repair, replace, augment or silence essentially any gene of interest in a target cell, opening up new areas of the proteome for drug targeting [12]. Other advantages of gene therapy versus small molecules include the ability to effect long-lasting clinical benefit with a single treatment and the potential to control cellular targeting via vector modifications [13].

Indeed, gene therapy is already making a clinical impact in the field of neurology, with Nusinersen, an antisense oligonucleotide therapy approved in Spinal muscular atrophy (SMA), and more recently Luxturna, a viral-based gene replacement strategy approved in Leber’s congenital amaurosis, acting as the first disease-modifying therapies in both of these diseases [14, 15]. In addition, a clinical trial in SMA by AveXis using systemic delivery of recombinant adeno-associated virus 9 (rAAV9) carrying a replacement SMN1 gene recently proved safe and efficacious in neonates [16]. On the other hand, gene therapies are clearly expensive in the short-term, with current therapies costing at least $500,000 per treatment, and thus remaining unaffordable in many healthcare systems (see ref [17] for a thorough economic analysis).

This review will highlight key targets for ASD gene therapy, the utility of ASD models, and recent advances in our ability to deliver such therapies to the central nervous system (CNS). It will then move on to discuss recent gene therapy strategies in ASD, concentrating on conditions with available preclinical data, and the roadblocks facing their clinical translation.

## Genetic targets in ASD

ASD may be divided into conditions driven by a single genetic defect (monogenic ASD) and conditions driven by multiple genetic defects (polygenic ASD). Monogenic ASD conditions often contain a variable cluster of phenotypes which include autism as part of a syndrome [18]. Whilst only accounting for ~ 5% of ASD cases [18], such disorders are prime candidates for gene therapy for two major reasons: firstly, they lend themselves to developing genetic models of ASD, which enable elucidation of the genotype to phenotype pathway, the potential for disease reversibility postnatally, and the efficacy/toxicity of novel therapeutics; secondly, correction of a single causative protein defect has the potential to arrest and possibly reverse disease pathology. Indeed, a basis for preclinical gene therapy studies in ASD was founded by identification of the nature and function of causative genes for a number of monogenic conditions with autistic features, including Rett syndrome (RS), fragile X syndrome (FXS), Angelman syndrome (AS) and tuberous sclerosis (TSC) [19–23] (Table 1).

**Table 1:** Genotypic and phenotypic characteristics of monogenic conditions with ASD features

Monogenic ASD	Mutated gene	Chromosome	Protein function	Autism prevalence	Other characteristics
Fragile X syndrome	FMR1 (encodes FMRP)	X	Binds and transports specific mRNAs from the nucleus to the ribosome [123]	~ 30% [124]	Long/narrow face, macroorchidism, long ears and philtrum, mild to moderate intellectual disability, hyperactivity, intellectual disability (ID), seizures
Rett syndrome	MECP2	X	Chromatin modification [125]	~ 60% [124]	Microcephaly, breathing irregularities, language deficits, repetitive/stereotyped hand movements, epilepsy, ID
MECP2 duplication syndrome	MECP2	X	Chromatin modification [125]	~ 100% [126]	Brachycephaly, spasticity, recurrent respiratory infections, gastrointestinal hypermotility, genitourinary abnormalities, epilepsy, ID
Tuberous sclerosis	TSC1 TSC2	9 16	Inhibition of translation via mTORC1 inhibition [127]	~ 50% [124]	Benign tumours in multiple organs, epilepsy
Angelman syndrome	UBE3A	15	Targeting of proteins for destruction via ubiquitin-tagging [41]	~ 30% [124]	Cheerful demeanour, microcephaly, epilepsy, speech deficits, sleep disturbance, epilepsy, ID

*Abbreviations*: *FMR1* fragile X mental retardation 1, *FMRP* fragile X mental retardation protein, *MECP2* methyl-CpG-binding protein 2, *TSC1* tuberous sclerosis 1, *TSC2* tuberous sclerosis 2, *UBE3A* ubiquitin-protein ligase E3A

**Fig. 1:**
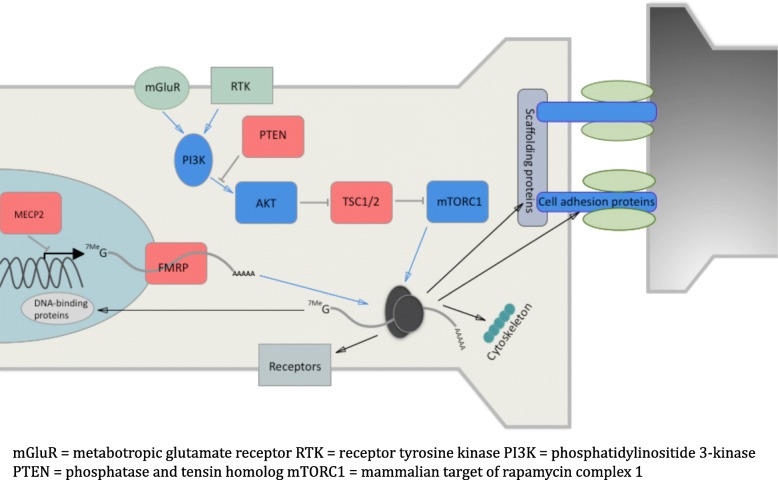
Proteins known to cause monogenic ASD are shown in red. Some of these, including TSC1/2, directly impact on ribosomal translation via the AKT-mTORC1 (mechanistic target of rapamycin complex 1) pathway, leading to altered synaptic protein expression and hence altered synaptic function. Others feed into this loop at the level of transcript production (MECP2 [125]) and selection (FMRP [123]) and protein degradation (UBE3A [128], not shown). Many other ASD-linked proteins also act within this synaptopathic loop, including various cell adhesion molecules (e.g. neuroligins [NLGNs], neurexins [NRXNs] [129, 130]), scaffolding proteins (e.g. postsynaptic density protein 95 [PSD95] [131]), cytoskeletal proteins (e.g. disrupted in schizophrenia 1 [DISC1] [132]), receptors (e.g. AMPA, NMDA, mGluR [133, 134]) and DNA-binding proteins (e.g. chromodomain-helicase-DNA-binding protein 8 [CHD8] [135, 136]). The entire rapidly expanding list of over 900 ASD-linked genes can be found at the Simons Foundation Autism Research Initiative (SFARI) database (https://gene.sfari.org/database/human-gene/)

More recently, our understanding of the genetic landscape of ASD has been revolutionised by several whole-exome and whole-genome sequencing studies, identifying hundreds of de novo and rare inherited variants influencing sporadic ASD risk [24–32]. Many of these genes appear to be involved, either directly or indirectly, in synaptic morphology and activity, leading to the concept of ASD as a ‘synaptopathy’ [33, 34] (Fig. 1). Certainly, the idea of using gene therapy to increase or decrease the expression of target proteins within this network and ‘retune’ the synapse is a powerful one, which may be applicable to certain ASD cases.

However, in such a heterogeneous condition as ASD, it is important not to become evangelical about a single causative mechanism, especially given recent insights into the apparently critical roles of immune dysfunction and epigenetics in at least certain ASD cases [35, 36]. Furthermore, recent phase II clinical trials which looked to regulate synaptic function via GABA and glutamate receptor modulation failed to demonstrate significant overall benefit, despite strongly positive responses in certain patients [37, 38]. Thus, it is important to consider whether targeting the synapse using gene therapy may be most appropriate for correcting particular ASD endophenotypes in specific patient subsets, rather than seeing it as a panacea for ASD, a topic that is returned to later in this article.

## Modelling ASD in rodents: a platform for proof-of-principle studies

The field of gene therapy is littered with examples of therapies which failed to translate from their preclinical promise. In many cases, blame can be attributed to the predictive validity of the animal model used, which is itself related to its construct validity (i.e. how well the model mimics disease aetiology) and face validity (i.e. how closely the model’s phenotype represents the human disorder) [39].

Given the challenges clinicians have faced in developing diagnostic criteria for ASD [40], and how various social traits often appear to be inherently ‘human’ qualities (although this is itself highly contested [41]), it is little wonder that generating ASD animal models with good face validity has been challenging. Nevertheless, whilst caution should always be taken when ascribing behavioural outcomes in animals to autism, various monogenic ASD rodent models have capably demonstrated quantifiable social and communicative behavioural traits [42–44], laying the ground for preclinical therapeutic studies.

As a caveat, it must be noted that a major limitation of ASD animal models relates to their inability to reflect heterogeneous environmental influences on the ASD phenotype, with various toxic, inflammatory and psychosocial factors difficult to incorporate in robust, reproducible animal models. This deficit in construct validity is especially relevant in modelling polygenic ASD, in which the gene × environment interaction plays a more fundamental role [45], and which therefore bears a particular risk for future translational work.

## Locating and reversing ASD pathophysiology

Identifying specific cells and circuits dysregulated in ASD is crucial in gene therapy design, as vectors may be targeted to specific brain regions or cell types (discussed in detail later). In particular, nascent data suggest the hippocampus, cerebellum and corpus callosum contain key pathogenic circuits [34, 46–48], whilst influential cell types include pyramidal cells, Purkinje cells and glial cells [34, 49].

Recent technological advances have begun to elucidate the relationship between cell type-specific function and particular ASD endophenotypes. For example, in a RS mouse model, cre/lox-mediated deletion of MECP2 specifically from forebrain glutamatergic neurons led to a partial disease phenotype, with deficits in social behaviour and motor coordination, but preserved locomotor activity and fear-conditioned learning [50]. Meanwhile, in a TSC mouse model, chemogenetic excitation of the Right Crus I (RCrusI) region of the cerebellum—an area consistently noted to be altered in the ASD brain in neuroanatomical and neuroimaging studies [51, 52]—was sufficient to specifically rescue social impairments, without rescuing repetitive or inflexible behaviours [53].

Within individual circuits, it appears that different ASD mutations may have opposing effects on synaptic function. For example, TSC2^+/−^ and FMR1^−/y^ knockouts appear to have opposite effects on mGluR-dependent long-term depression (LTD) in the hippocampus, whilst mice bred with both mutations balance each other out at the synaptic and behavioural levels [54]. Such data not only exemplify the heterogeneic nature of ASD but also highlight the necessity of optimal synaptic control, and are the first hint that a successful gene therapy must walk a narrow therapeutic tightrope between over- and under-stimulating synaptic transmissions.

A crucial further question is whether autistic phenotypes can be reversed or are neurodevelopmentally fixed. Remarkably, an array of studies in different monogenic ASD animal models have consistently demonstrated the potential for reversal of established neuronal dysfunction, either after pharmacological intervention or genetic reactivation of silenced alleles [55–60]. These findings imply that postnatally, indeed post-symptomatically, the genetic horse may not yet have bolted, and genetic correction via a delivered vector might be useful in treating cognitive dysfunction.

## Delivering gene therapies to the CNS

If the ASD phenotype is reversible rather than neurodevelopmentally fixed, as implied by studies in monogenic animal models, then it follows that continuous genetic correction will be necessary for a sustained therapeutic effect. Currently, only viral packaging systems have combined efficient transduction with long-term gene expression in vivo [61] (although, as will be discussed later, certain ASD conditions may be amenable to non-virally delivered antisense nucleic acid therapies).

Of the viruses which can transduce post-mitotic cells, rAAVs have emerged as the principal CNS delivery candidate [62]. This is based upon their relatively low immunogenicity (compared to adenovirus and herpes simplex virus), limiting the likelihood of an encephalitic immune response, their ability to persist in episomal form, reducing their oncogenic potential (compared to retroviruses [63, 64]), and their high production titres [12]. Indeed, rAAV vectors have already been used safely in a number of early clinical studies in CNS gene therapy, in disorders ranging from SMA to idiopathic Alzheimer’s disease [65].

Optimising cell-specific targeting is critical in maximising the number of transduced target cells/dose and limiting off-target toxicity. There are two major ways in which the spatial dynamics of rAAV vectors can be adjusted. Firstly, the properties of the vector itself can be modified, to include a cell type-selective capsid (e.g. AAV9 is particularly neurotropic [66, 67]) and/or promoter [68].

Secondly, the mode of delivery can be adjusted. Historically, rAAV vectors have been delivered intraparenchymally via stereotactic CNS injection, leading to high local concentrations with limited vector spread [69]. Although invasive (each injection requiring a craniotomy), such a localising method of delivery might have utility in correcting specific dysregulated ASD circuits linked to particular clinical endophenotypes, analogous to the recent improvement in motor scores seen after lentiviral vector delivery of a dopamine-producing gene therapy to the nigrostriatal pathway in Parkinson’s disease [70].

However, ASD appears to involve global synaptic dysregulation and thus will require global CNS gene correction to fully reverse cognitive phenotypes [71, 72]. The discovery that rAAV9 crosses the blood-brain barrier (BBB) and globally transduces CNS neurons and glial cells [73], and the recent derivation of more efficient BBB-traversing rAAVs by targeted evolution [74], has opened up the possibility of using intravenous injection in ASD gene therapy.

It remains to be seen, however, whether side effects relating to peripheral tissue transduction, as well as the presence of neutralising circulating antibodies (anti-AAV9 antibodies are present in 47% of humans), will preclude intravenous administration in various ASDs [75–77]. Changes to the viral vector nucleic acid sequence outside of the transgene—such as the inclusion of ‘detargeting’ sequences recognised by micro RNAs (miRNAs) expressed specifically in off-target cells [78]—might circumvent the former issue, but use up highly limited space (rAAV’s packaging capacity is limited to ~ 5 kb [79]). The latter issue may be negotiated by the use of engineered rAAV capsids, which may have lower neutralising antibody seropravalences [80].

An alternative to systemic delivery is intrathecal administration, which potentially combines (relatively) safe administration and global CNS transduction with fewer peripheral complications, and a higher spatial resolution limiting the dose requirements. However, there are conflicting data regarding the ability of intrathecally delivered rAAV to efficiently transduce areas outside the spinal cord [81], as well its own ability to avoid both peripheral leakage [82] and a neutralising antibody response [83, 84]. Of note, an intrathecal AAV9 approach has been corrective in a model of giant axonal neuropathy [85] and has progressed to a clinical trial.

## Gene therapy strategies in monogenic ASD

### Gene replacement

In ASD disorders defined by loss of function mutations (e.g. RS, FXS, TSC), simple gene replacement may restore synaptic function [12]. Given the limitations imposed by imperfect gene delivery strategies, a key question is whether sufficient transduction of target cell types can be attained to exert phenotypic benefit.

Encouragingly, a number of studies using monogenic animal models have demonstrated behavioural improvements after rAAV-delivered gene replacement. In a RS mouse model, systemic delivery of a rAAV9-MECP2 vector sufficient for ~ 10% CNS transduction (of principally neuronal cells) led to moderate behavioural improvements [86, 87]. Meanwhile, at an ~ 6-fold higher vector dose, ~ 25% CNS transduction resulted in marked behavioural and phenotypic improvements [88]. Finally, it was recently demonstrated that rAAV-mediated delivery of even a fragment of the MECP2 gene (lacking N- and C- terminal regions along with a central domain) led to phenotypic improvement in RS mice, potentially allowing extra room for construct modifications to aid target cell transduction and expression [89]. Similarly, substantial phenotypic improvements were seen in studies using FXS and TSC models after intra-CNS delivery of replacement genes, although none of these studies quantified CNS transduction [90–92].

Although a cause for optimism, none of the above studies evidenced total phenotypic reversal after gene replacement. Such incomplete phenotypic reversal may be secondary to insufficient CNS transduction. In RS for example, ~ 80% gene reactivation in neuronal cells appears to be sufficient and necessary for total phenotypic reversal [56, 93]. However, increasing the vector dose in order to increase transduction must be balanced against the risk of dose-related toxicity. This may occur secondary to off-target cell transgene expression: for example, transgene-specific liver toxicity was seen at high doses of rAAV9-MECP2 [86, 94], possibly due to MECP2’s role in liver metabolism [95].

Toxicity may also occur secondary to supraphysiological expression in target cells. For example, after rAAV-mediated delivery of FMRP in FXS, toxicity developed at 2.5-fold expression above wild type [96], whilst duplication of MECP2 leads to MECP2 duplication syndrome in males [97–99]. Such toxicity may occur even at low transduction percentages due to uneven vector distribution within the CNS or, in the case of X-linked disorders in females, due to a mosaic pattern of CNS expression caused by random X-inactivation [100]. Reassuringly, a fragmented version of the MECP2 promoter appeared to limit MECP2 expression to physiological levels in both wild type and MECP2^null/x^ female mice, even at vector doses leading to ~ 25% CNS transduction [88].

Nonetheless, further studies are required to pinpoint the optimum balance between CNS transduction and on-target toxicity in various ASD syndromes. Additionally, future gene replacement studies must better characterise the relationship between gene dose and dendritic function (which was not assessed in any of the above studies).

### RNA knockdown

Gene expression can be silenced by sequence-specific knockdown of mRNA transcripts using techniques such as antisense oligonucleotides (ASOs) and short interfering RNAs (siRNAs), which use the exquisite specificity conferred by Watson-Crick base pairing to bind particular mRNA transcripts and prevent their translation (for a detailed mechanism see ref [101]). These nucleic acids are typically relatively easy to manufacture, can be modified to limit degradation and inflammation, and do not require a viral vector (although long-term expression of ASOs is possible using viral delivery of short hairpin RNAs [shRNAs]) [101, 102]. Indeed, such therapies are already being used in the treatment of SMA and in clinical trials for Huntington’s disease [103, 104].

These techniques are principally useful when total or partial knockdown of a particular transcript may restore synaptic function. For example in MECP2 duplication syndrome, halving MECP2 expression was shown to restore cellular function and phenotype postnatally in a conditional MECP2-overexpressing mouse model [105]. In the same study, intraventricular delivery of ASOs (delivered at a constant rate by a pump) specifically targeting MECP2 led to widespread ASO distribution in the CNS, effective knockdown of MECP2 to nearly wild type levels, and sustained phenotypic reversal (~ 10 weeks) [105].

Another strategy in which RNA silencing may be useful is in knocking down a gene which inhibits a target gene’s expression, i.e. disinhibition. For example, triplication of 15q11-13 leads to a relatively common and highly penetrant type of autism linked to increased expression of UBE3A (which functions as a transcription regulator in addition to its ubiquitin ligase function) and subsequent downregulation of Cerebellin 1 Precursor (Cbln1), a synaptic organising protein, in the ventral tegmental area (VTA) [106]. Thus, knockdown of UBE3A could be used to restore sufficient Cbln1 expression in the VTA, which has already been shown to effect phenotypic change after cre/lox-mediated restoration in a UBE3A-triplicated mouse model [106].

Another application of this strategy could be in AS, where the long non-coding UBE3A antisense transcript (UBE3A-AST) causes imprinting of the paternal UBE3A allele, ensuring that a loss of maternal UBE3A allele function yields the AS phenotype (another example of how genetic defects in ASD may be bidirectional, with optimal gene expression in specific brain regions crucial). Indeed, a recent paper demonstrated that a single intracerebroventricular injection of a degradation-resistant ASO targeting UBE3A-AST in an adult AS mouse model led to specific and sustained reductions in UBE3A-AST levels, with partial restoration (~ 40%) of UBE3A levels throughout the CNS [107].

Interestingly, in the same study, whilst motor deficits were restored, other behaviours—such as anxiety and repetitive behaviours—were not. A later study, using Cre-dependent UBE3A reactivation in an AS mouse model, showed a temporal dependence for specific phenotype reversal, with anxiety and repetitive behaviours requiring gene reactivation during early development, whilst motor deficits could be restored into adulthood [108]. Such temporal factors have not been thoroughly investigated in other monogenic ASDs but are clearly critical when considering the time point of useful intervention in humans.

Finally, in a similar vein to excessive gene replacement, hyper-knockdown of target RNA may lead to rebound toxicity in both target cells and off-target cells. Furthermore, both ASOs and siRNA may cause unpredictable off-target knockdown [109]. From this perspective, the requirement for regular intra-CNS administration of ASOs is a double-edged sword in ASD: whilst on the one hand, it is clearly less convenient than a once-off injection of viral vector, on the other hand, it allows for the possibility of dose uptitration and determination of an optimal therapeutic level.

### Gene editing

One of the most exciting recent developments in gene therapy is the advent of easily customised sequence-specific editing techniques, such as CRISPR (clustered regularly interspaced short palindromic repeats)-Cas9, enabling either correction of a genetic mutation via non-homologous recombination (providing there is a suitable template) or gene silencing via non-homologous end-joining [110]. Such techniques would generally enable gene expression at physiological levels in target cells, negating the problems of transgene-associated toxicity seen with both gene replacement and RNA knockdown techniques.

Unfortunately, at least in vivo, gene editing techniques still remain a distant therapeutic prospect, with a wealth of technical hurdles to overcome, including how to deliver gene editing systems to target cells, how to increase the efficiency of editing, and how to avoid off-target editing [111, 112]. Still, recent work by Doudna and colleagues provides optimism in this regard, with the demonstration of greatly improved editing of post-mitotic neurons in adult mouse brains using cell-penetrating peptides tagged onto Cas9 ribonucleoprotein complexes [113].

## Prospects for gene therapy in polygenic ASD

As previously mentioned, in comparison with monogenic ASD, polygenic ASD has a greater environmental component driving its phenotype [45]. In this respect, damaging, nonsynonymous postzygotic mutations in whole-exome sequences from the largest collection of trios with ASD were recently identified, with some of these genes being particularly enriched for expression in the amygdala, a key brain region for social conditioning and learning [114]. Such factors, combined with the current paucity and constructional limitations of animal models in polygenic ASD, make it a less obvious target for gene therapy.

Nonetheless, despite the bewildering array of rare genetic mutations linked to polygenic ASD, an important focus of these mutations appears to be in the regulation of synaptic function, with diverse ASD mutations potentially connecting aberrant translational inputs and outputs (Fig. 1) [34]. For example, in mice, deletion of the translational repressor Eukaryotic translation initiation factor 4E-binding protein 2 (4E-BP2) led to overexpression of the NLGN class of cell adhesion molecules [115], mutations of which have been causally linked to ASD [46, 116, 117]. Furthermore, such deletion resulted in disruption of the ratio of excitatory to inhibitory synaptic inputs, as well as an ASD behavioural phenotype, which was corrected by NLGN1 knockdown [115].

This leads to the idea that many ASD mutations might be treated by fine-tuning the expression of influential proteins acting within dynamic translational loops. The apparently fundamental role of the PI3K-AKT-mTOR pathway in various causes of monogenic ASD [118], as well as the phenotypic reversal seen using small molecule inhibitors of mTORC1 preclinically [119], suggests that this pathway may be a critical target for gene therapy in certain cases of ASD.

However, given ASD’s heterogeneity, it is once again important not to focus myopically on a single pathway. Rather, instead of embarking on a ‘one size fits all’ therapeutic approach, the effect of any particular ASD mutation on translational output and synaptic function should be categorised, before deciding whether and how to target a particular gene or pathway. For instance, NLGN3 knockout mice demonstrate a FXS-like disruption of mGluR-dependent synaptic plasticity [120], suggesting that either FMRP overexpression or PI3K-AKT-mTOR pathway knockdown (given the aforementioned opposition of these two pathways) might correct this phenotype.

Finally, recent evidence has emerged of ASD behaviours caused by amino acid deficits [121, 122]. For example, homozygous dysfunction of the BBB solute carrier transporter 7a5 (SLC7A5) and corresponding CNS loss of branched chain amino acids (BCAAs) has been linked to ASD, which is reversible in mouse models upon intra-CNS administration of BCAAs [122]. Thus, direct protein replacement therapy might provide an important additional therapeutic avenue in certain ASD cases. It is also possible to imagine using gene therapy as an adjunct here: for example, combining systemic BCAA replacement with vector-delivered SLC7A5 targeting BBB cells.

## Conclusions

Given the heritable component of ASD, gene therapy offers a promising alternative to conventional small molecule therapies. Preclinical studies over the last 5 years using animal models displaying autism-like traits have demonstrated that directly altering gene expression using rAAV-delivered transgenes can reverse the behavioural phenotype, either via gene replacement or RNA knockdown. Such studies establish proof-of-concept and set up a platform for clinical translation in various monogenic ASDs, with RS being a frontrunner in this regard.

However, major hurdles remain, not least the fact that the majority of ASD disorders, even monogenic ones, show variable penetrance, with epistatic and gene × environment interactions determining phenotype. Not only is such genetic and environmental heterogeneity inherently difficult to model, hindering clinical translation, but also in clinical trials that do go ahead, ASD subgroups that benefit from a particular treatment may be lost amongst other unsuitable subgroups. Furthermore, we still do not know whether, or in which cases, epigenetic factors may preclude reversibility in humans. Cyclically, this brings us back to the question of animal models and whether these have sufficient construct validity to actually begin to answer such questions in the first place.

A number of additional questions remain: Firstly, can vector design be optimised to the extent that intravenous delivery achieves sufficient CNS transduction without peripheral toxicity? Secondly, where is the optimum balance between CNS transduction and the risk of on-target transgene-related toxicity for each ASD syndrome? Thirdly, will demonstrations of acceptable levels of CNS toxicity hold when studies commence in larger animal models? Fourthly, is there a time point beyond which some or all autistic features lose their reversibility? Answering these questions will be key to moving ASD gene therapy into clinical trials, and perhaps one day generating a genetic treatment for ASD.

## Abbreviations

AS: Angelman syndrome
ASD: Autistic spectrum disorder
ASO: Antisense oligonucleotide
BBB: Blood-brain barrier
BCAA: Branched chain amino acids
Cbln1: Cerebellin 1 precursor
CHD8: Chromodomain-helicase-DNA-binding protein 8
CNS: Central nervous system
CRISPR: Clustered regularly interspaced short palindromic repeats
DISC1: Disrupted in schizophrenia 1
FMR1: Fragile X mental retardation 1
FMRP: Fragile X mental retardation protein
FXS: Fragile X syndrome
LTD: Long-term depression
MECP2: Methyl-CpG-binding protein 2
mGluR: Metabotropic glutamate receptor
miRNA: MicroRNA
mTORC1: Mammalian target of rapamycin complex 1
NLGN: Neuroligin
NRXN: Neurexin
PI3K: Phosphatidylinositide 3-kinase
PSD95: Postsynaptic density protein 95
PTEN: Phosphatase and tensin homolog
rAAV: Recombinant adeno-associated virus
RCrusI: Right Crus I
RS: Rett syndrome
RTK: Receptor tyrosine kinase
SFARI: Simons Foundation Autism Research Initiative
shRNA: Short hairpin RNA
siRNA: Short interfering RNA
SLC7A5: Solute carrier transporter 7a5
SMA: Spinal muscular atrophy
TSC: Tuberous Sclerosis
TSC1: Tuberous sclerosis 1
TSC2: Tuberous sclerosis 2
UBE3A: Ubiquitin-protein ligase E3A
VTA: Ventral tegmental area
